# Candidate Transcriptomic Sources of Inbreeding Depression in *Drosophila melanogaster*

**DOI:** 10.1371/journal.pone.0070067

**Published:** 2013-07-29

**Authors:** Carlos Garcia, Victoria Avila, Humberto Quesada, Armando Caballero

**Affiliations:** 1 Departamento de Xenética, Universidade de Santiago de Compostela, Santiago de Compostela, Galicia, Spain; 2 Institute of Evolutionary Biology, University of Edinburgh, Edinburgh, Scotland, United Kingdom; 3 Dep. Bioquímica, Genética e Inmunología, Universidade de Vigo, Vigo, Galicia, Spain; University of Houston, United States of America

## Abstract

The genomic causes of inbreeding depression are poorly known. Several studies have found widespread transcriptomic alterations in inbred organisms, but it remains unclear which of these alterations are causes of the depression and which are mere responses to the ensuing physiological stress induced by increased homozygosity due to inbreeding. Attempting to differentiate causes from responses, we made a c-DNA microarray analysis of inbreeding depression in *Drosophila melanogaster*. The rationale of the experiment was that, while depression is a general phenomenon involving reductions in fitness in different inbred lines, its first genetic causes would be different for each inbred line, as they are expected to be caused by the fixation of rare deleterious genes. We took four sets of inbred sublines, each set descending from a different founding pair obtained from a large outbred stock, and compared the expression of the three most depressed sublines and the three least depressed sublines from each set. Many changes in expression were common to all sets, but fourteen genes, grouped in four expression clusters, showed strong set-specific changes, and were therefore possible candidates to be sources of the inbreeding depression observed.

## Introduction

The consequences of the increase in homozygosity due to mating between close relatives (inbreeding) are well known in the evolutionary [1], animal and plant breeding [2], and conservation [3] contexts. Inbreeding is responsible for the reduction in the performance of reproductive traits (inbreeding depression) with important implications on the capacity of populations to evolve and adapt to new environmental changes and, eventually, in their long-term viability [4]. However, the genomics of inbreeding depression is not well understood [5]. Knowing the mechanisms involved in the causation of inbreeding depression at the genomic level would enable the identification of optimum reproducing individuals in conservation and genetic improvement plans [6]–[8], and to predict the effectiveness of purging measures [9]–[11]. Of special interest would be to measure the impact of the variation in gene expression on the magnitude of inbreeding depression, because experimental evidence has shown that most phenotypic differentiation, both between species [12]–[14] and within populations [15] (reviewed in [16]) is related with changes in gene regulation.

Some whole-genome c-DNA microarray-based transcriptomic comparisons between inbred and outbred lines are already available for *Drosophila melanogaster*. Kristensen *et al*. [17] found that their inbred lines changed the regulation of many different genes, especially those related with metabolism, biological defence and stress responses. Also using microarrays in this species, Ayroles *et al*. [18] compared the transcriptome of lines completely homozygous for different third chromosomes derived from a wild population and showing strong and weak depression for male competitive reproductive success. Again, many genes changed their expression between depression levels and those related with basic metabolism, stress and defence responses were overrepresented. In a previous paper [19] (see also [20]), we have presented the results of a new analysis where both inbreeding and inbreeding depression effects on gene expression are analysed. As in previous studies, we found that inbreeding caused large-scale changes in gene expression. But, in addition, we observed that for most of the genes with a change in expression under inbreeding, the least depressed sublines were those showing the largest departures in expression from that of the outbred control. This pattern was consistent with a protective role of expression changes against inbreeding effects. Thus, gene expression would change in a complex way under inbreeding. On the one hand, the defective regulation of some genes would contribute to the depression, whereas on the other, many functional expression adjustments are assumed to maintain fitness in the new genetic situation.

These overall transcriptomic responses to inbreeding detected in joint analyses of several lines provide ample information about the development and dynamics of the depression, but not so much about its first genetic causes. According to population genetics studies, the most important source of inbreeding depression in many species is homozygosity for rare, deleterious and, at least partially, recessive genes [21]–[29]. Because they are rare, each of these deleterious genes is unlikely to be present in many families, and thus, those genes homozygous and originally causing depression in different inbred lines of a population are expected to be different [4]. These genes could build up a signal common to several inbred lines if they had cascade effects in progressively wider gene networks, which would eventually overlap between lines and result in a whole-experiment response. For example, the malfunctioning of a given gene pathway might be due to the defective regulation of any of the genes involved in the pathway and thus have different causes in different inbred lines, but trigger the same compensating transcription response across the genome. Similar large-scale changes in transcription would thus be observed in many different inbred lines [5]; [30]–[31]. Thus, it may be reasoned that, among the changes in expression associated with inbreeding depression, those observed in one single line are more likely to be related to the original causes of depression, whereas those common to many different lines would be unspecific responses to the overall depression conditions.

In this paper we present an analysis of the transcriptome of *Drosophila melanogaster* aimed at trying to find a set of candidate genes responsible for inbreeding depression. The approach consists of looking for changes in gene expression both associated with depression and line specific, and thus resulting from putative genetic alterations originally causing the depression. We found several gene expression changes fulfilling these conditions and being therefore candidates for transcription sources of inbreeding depression.

## Materials and Methods

### Experimental Design

**Figure 1:**
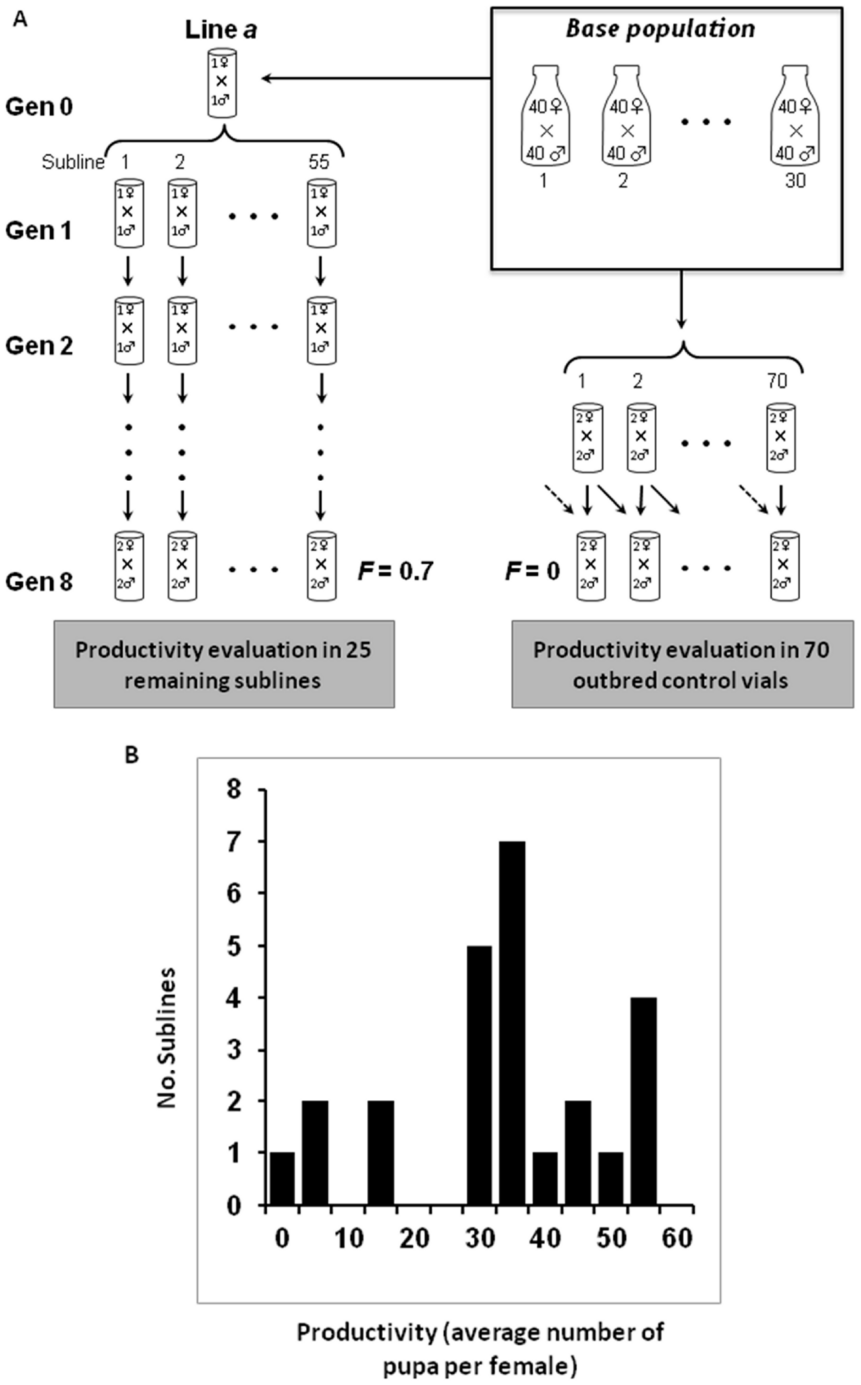
Experimental lines and sublines. (A) Schematic representation of the experimental design for one of the four inbred lines (line *a*) and for the outbred control. 55 inbred sublines were established and maintained by sib mating for eight generations. Pupa productivity was evaluated in the remaining sublines at generation 8. Pupa productivity was also simultaneously evaluated from 70 replicated vials of an outbred control. (B) Distribution of average productivities (number of pupa per female 14 days after mating across three replicated vials) for the 25 sublines of line *a* remaining at generation 8.

The biological material used in this work is described in [19], where a detailed description of the experimental design and main analytical tools employed can be found. Briefly, the base population was founded in November 2006 from a large sample (>1000 females) collected in a wine cellar close to Vigo (Galicia, NW Spain). This population was maintained in 30 bottles with about 80 individuals per bottle with circular mixing of bottles each generation until the start of the experiment in July 2008 (Figure 1A). From this population we sampled four couples to found four inbred lines (*a*, *b*, *c*, *d*). Figure 1A illustrates the experimental procedure followed for each of the lines. From each initial couple, 55 inbred sublines were established and maintained for 8 generations of sib mating with the objective of fixing in different sublines the genetic variation segregating in the initial couple. Single pair mating was carried out until generation 4, but two males and two females were mated per vial thereafter, to avoid further subline losses. The final average inbreeding coefficient was about 0.7. At generation 8, the number of remaining sublines was 25, 31, 17 and 27 for lines *a*, *b*, *c* and *d*, respectively. Pupa productivity (total number of progeny produced per female after 14 days) was evaluated in three replicated vials per subline from generation 8. Pupa productivity was also evaluated simultaneously in 70 replicated vials of an outbred control obtained from the base population as illustrated in Figure 1A. Figure 1B shows the distribution of average productivities per female (across three replicates) for the 25 sublines of line *a* at generation 8.

**Figure 2:**
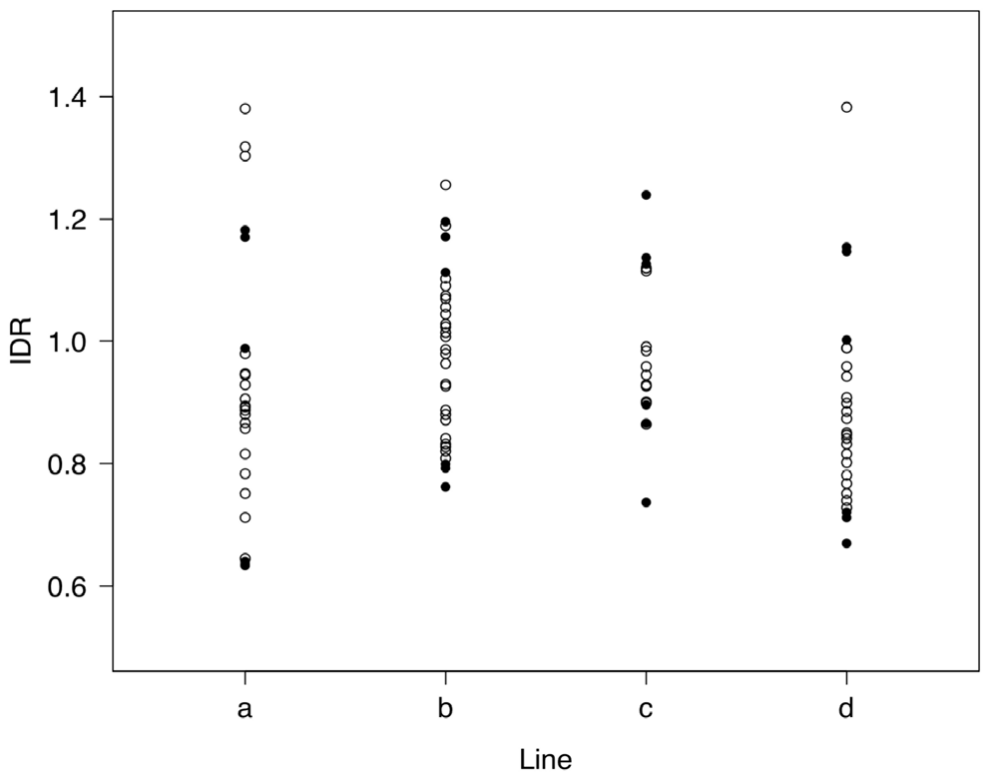
Inbreeding depression rate for productivity (IDR). IDR expressed in % per 1% increase in inbreeding coefficient for each of the sublines of the four inbred lines (*a*, *b*, *c*, *d*) at generation 8. The three sublines with the largest (provided enough individuals were available for analysis) and the lowest inbreeding depression in each inbred line chosen for expression analysis are shown with black symbols.

The average female productivity of the outbred control was *W_O_* = 101.97±2.39, whereas that for all inbred sublines was *W_I_* = 34.28±1.21. Thus, the overall inbreeding depression rate, obtained as (*W_O_*–*W_I_*)/(0.7 × *W_O_*), was about 1% per 1% increase in inbreeding. However, a substantial variation in inbreeding depression occurred among sublines of the same line (ranging from 0.64% to 1.4%). Figure 2 shows the inbreeding depression rates of each particular subline for all four lines.

### Analysis of RNA Expression

The three sublines with the largest productivity (lowest inbreeding depression) and the three sublines with the lowest productivity–but with enough individuals to be further analysed–(highest inbreeding depression), were chosen from each inbred line (see Figure 2) for a gene expression analysis using the Affymetrix Drosophila Genechip Array 2.0. Three replicates of the outbred control were also randomly chosen for analysis. For each sample, pools of 30 males from each selected inbred subline and from the outbred controls were used for total RNA extraction and hybridization with the array as described in [19]. We only analysed gene expressions in males to avoid the source of additional experimental variance associated to post-mating gene expression changes in females [32], or to different stages of egg development in pregnant females [33]. The Robust Multichip Average (RMA) method [34] was used for background adjustment, quantile normalization, and probe-level summarization of the microarray samples. RMA expression summary was computed using Partek Genomics Suite v. 7.3.3 (Partek) and the Affy package in Bioconductor [35]. To exclude genes that were not accurately detected in the data analysis probe, we retained for data analysis only the 9133 genes having at least one present call within at least one of the samples. The total number of arrays analysed was thus 27, i.e. 3 sublines with the largest depression and 3 sublines with the lowest depression for each of 4 inbred lines, plus 3 samples from an outbred control. The average variance between sublines expressions within lines was 0.028 for the control samples, 0.044 for the least depressed sublines, and 0.086 for the most depressed sublines [19]. The microarray data reported in this paper is available in the Gene Expression Omnibus (GEO) database under the accession number GSE47176.

### Identification of Candidate Genes Responsible for Inbreeding Depression

**Figure 3:**
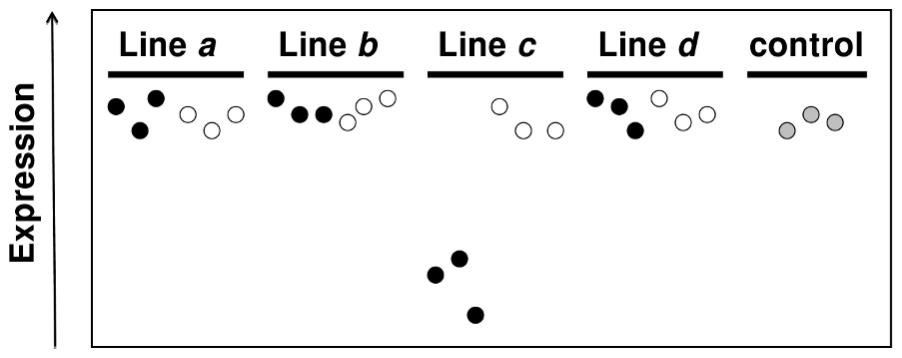
Schematic representation of the rationale behind the detection of *single line–level of depression* (SL-LD) *outliers*. The three most depressed sublines of line *c* are assumed to have a level of gene expression substantially lower than that from all other samples. This would be consistent with the existence of line-specific genetic alterations affecting both fitness and gene expression.

The genetic alterations causing inbreeding depression are expected to occur in particular sublines of a single inbred line. Thus, our objective was to look for individual probe sets (genes) whose change in expression because of inbreeding was restricted to a given depression level (most depressed or least depressed) within a single line. Figure 3 illustrates the main idea behind this procedure. In this schematic representation, the three most depressed sublines of line *c* have a level of gene expression substantially lower than that from all other samples. We call these outliers from the same line and depression level *single line–level of depression* (SL-LD henceforth) *outliers*, which would be consistent with the existence of line-specific genetic alterations affecting both fitness and gene expression.

To identify probe sets with large changes in expression in particular sublines we used the statistic of the Grubb’s tests for outliers [36]. This (also called extreme studentized deviate test) is a general purpose test for the detection of outliers [37]. The tests were applied in a sequential way as follows. First, for those probes having one outlier (i.e. a subline showing a significant change in expression) among the 24 inbred sublines, we analysed the remaining sublines to find a second outlier, and so on. Then we looked for probe sets having outliers belonging to the same line (one of four inbred lines) and depression level (most depressed or least depressed sublines).

The number of SL-LD outliers detected was contrasted with the expected number at random using the Westfall and Young’s [38] randomization procedure (10000 replicates; outbred control samples were not randomized). This multiple testing procedure takes into account the non-independence of the probe set expressions in the microarrays by comparing the number of positive test results observed with that obtained by randomizing the identification codes of the experimental units (see [39]). Each randomization for line and depression level codes was simultaneously applied to all probe sets showing two outliers, so that the codes were randomized while conserving the correlation structure of the list. We calculated the significance of our results by counting the number of randomization replicates that resulted in as many SL-LD outliers as observed in each direction of outlier expression.

To obtain functional annotations for the found outlier probe sets we used the DAVID Bioinformatics Resources 6.7 (http://david.abcc.ncifcrf.gov/home.jsp; [40]). These programs can use lists of probe sets as input, and provide results in terms of the corresponding genes.

### Identification of Sequence Mismatches

A possible problem with gene-expression measurements using microarray data is the occurrence of sequence mismatches due to sequence heterogeneity among target DNA at many base pairs. We evaluated this possibility by sequencing transcripts at target sequences for a subset of candidate genes to see which samples contained SNPs within them. Synthesis of cDNA from total RNA extractions was performed using the SuperScript VILO cDNA Synthesis kit (Invitrogen, Carlsbad, CA, USA) following the conditions recommended by the manufacturer. PCR primers for the amplification of a genomic stretch encompassing the DNA sequence from which the Affymetrix oligonucleotide probes are selected were designed using the *D. melanogaster* sequence (Flybase release 3.1). Primers and conditions for PCR amplification are given in Table S1. After purification of PCR products with NucleoSpin-PCR Clean-up columns (Macherey*-*Nagel, Düren, Germany), each PCR product was cycle sequenced in both forward and reverse directions with the same primers used for amplification. Sequences were assembled with the Seqman 7.0 program (DNASTAR; Madison, WI, USA). DNA sequences were multiple aligned against the microarray oligonucleotide match probes using the Clustal X 2.0 program [41] and further edited with the Bioedit 7.1.3.0 program [42]. IUPAC ambiguity codes were used for polymorphic sites. A site was designed polymorphic when more than one peak was present in the electropherogram and the weakest signal reached at least 50% of the strength of the strongest signal. To minimize the inclusion of bad reads as polymorphisms, we added the restriction that double peaks had to occur on the same position on both forward and reverse strands. The DnaSp 5.0 [43] and MEGA 5.1 [44] programs were used to estimate genetic differences among samples and microarray oligonucleotide probes. Newly reported DNA sequences were deposited in the EMBL nucleotide sequence database under accession numbers HG004127–HG004162.

## Results

**Figure 4:**
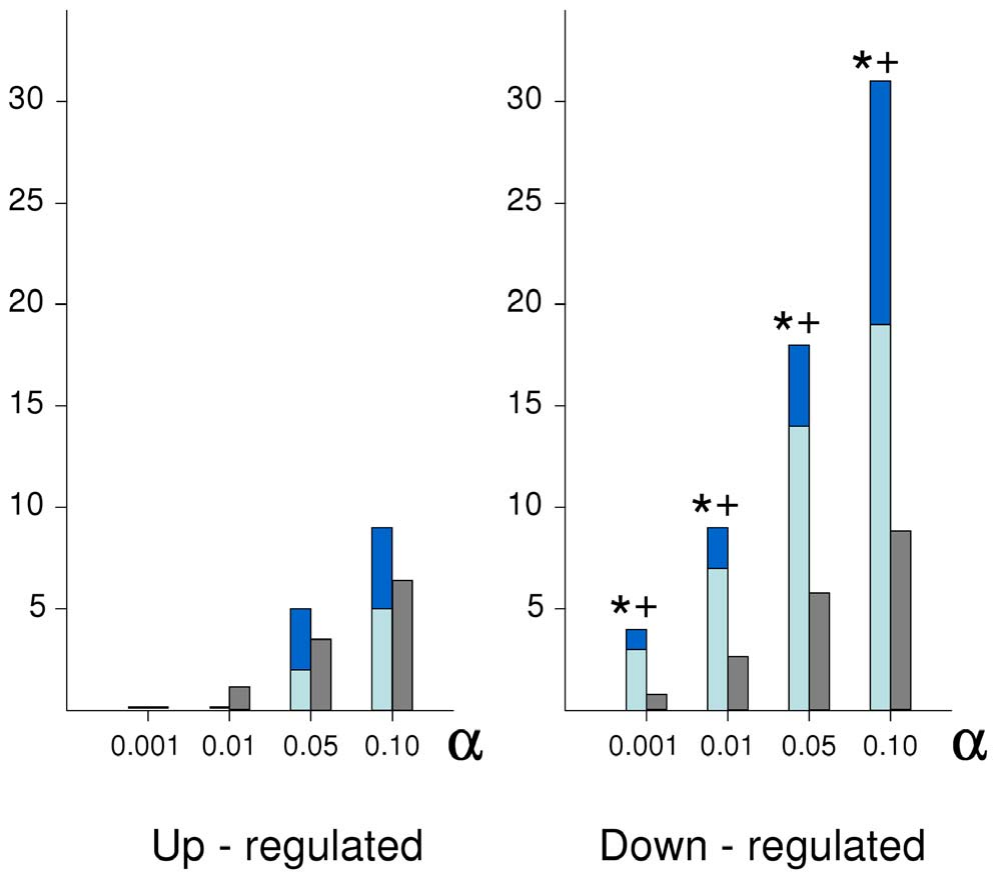
Analysis of probe sets having two outlier sublines in the same direction. We show the observed numbers of SL-LD outliers (dark and light blue, least and most depressed sublines, respectively), and the corresponding expected numbers (grey) after randomizing the line and depression level of the probe sets showing two up-regulated and two down-regulated outliers, respectively. Results are shown for decreasing *α* (probability of significance) values in the Grubb’s test to detect extreme expressions restricted to two sublines. The expected SL-LD numbers are not separated into least and most depressed sublines because their expected frequencies are equal after the randomization of line and depression level codes. *, *P*<0.05 in a randomization test (*n* = 10000) comparing observed and expected numbers; ^+^, *P*<0.05 in tests comparing the whole expected number in each class with that observed in the most depressed sublines only.

From the 9133 probe sets analysed we found very few having outlier expression in three sublines. For a level of significance of *α* = 0.05, only 9 probe sets had outlier over expression in three sublines, and 24 had outlier down expression in three sublines. In none of them the three outliers corresponded to the same line and depression level (i.e. they were not SL-DL outliers). Only one probe set fulfilled this condition when the *α* level was set to 0.1. Thus, next we looked for probe sets in which the two most extreme expressions in each direction were SL-DL outliers according to decreasing *α* values in the Grubb’s test. The results of this search are given in Figure 4; more detailed information is given in Table S2. The SL-DL outliers showing over-expression (up-regulated) were not significantly different from those expected at random for any value of *α*. However, there was a clear excess of SL-DL outliers in the sublines showing under expression (down-regulated). The most depressed sublines were the source of this excess. We thus focused on these probe sets showing down-regulated SL-DL outliers in the most depressed sublines for further analysis. In particular, we took the 14 probe sets detected using *α* = 0.05 in the Grubb’s test. We identify them henceforth by their corresponding gene code.

**Figure 5:**
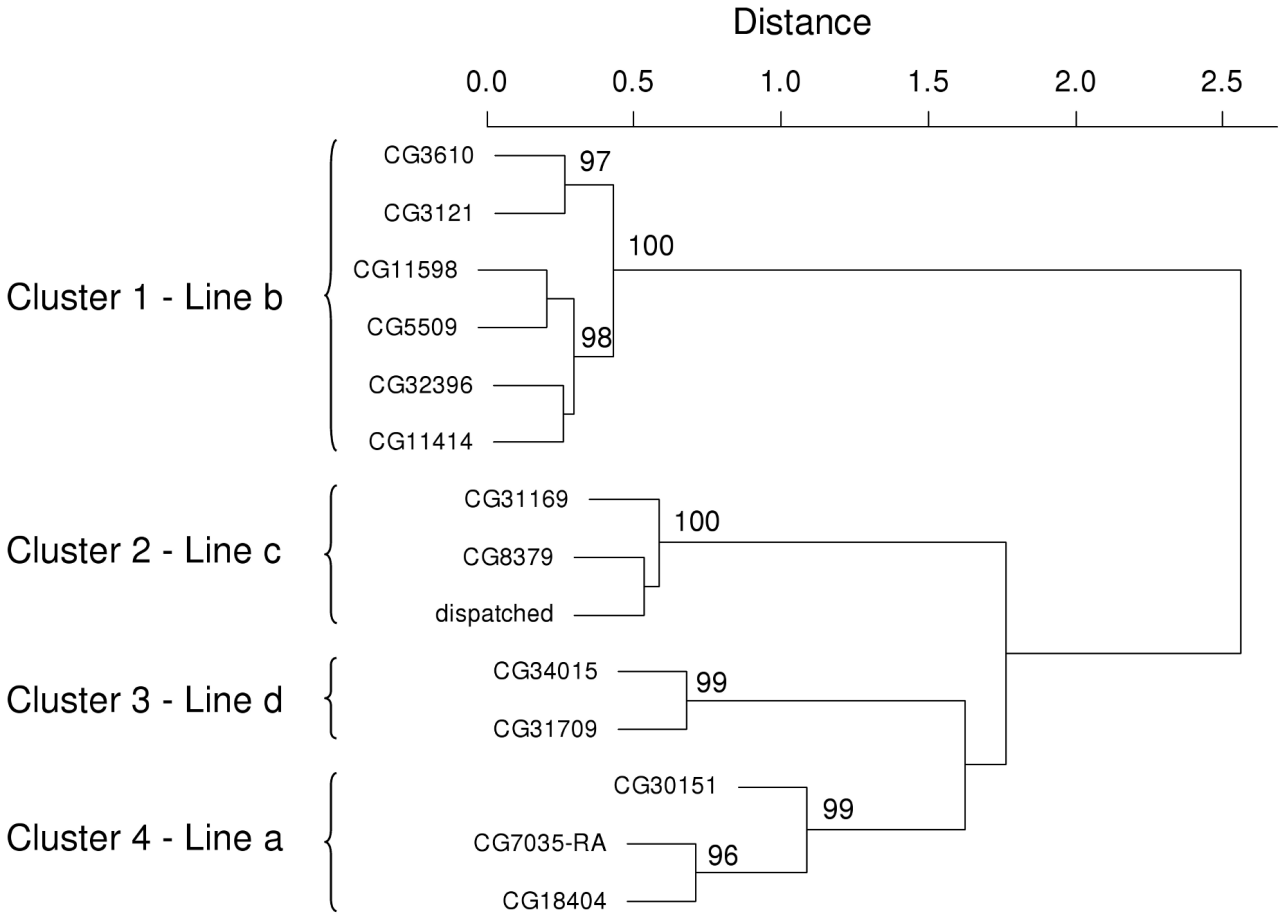
Hierarchical clustering of the 14 genes candidates to be sources of inbreeding depression. R [55] hclust function using “complete distance” as clustering method and absolute correlations as distance measure. The numbers in cluster splits are “approximately unbiased *p*-values” calculated by multiscale bootstrap resampling (1000 replications) using the pvclust function from the pvclust R package [56]. Only *p*-values higher than 95% are shown.

**Figure 6:**
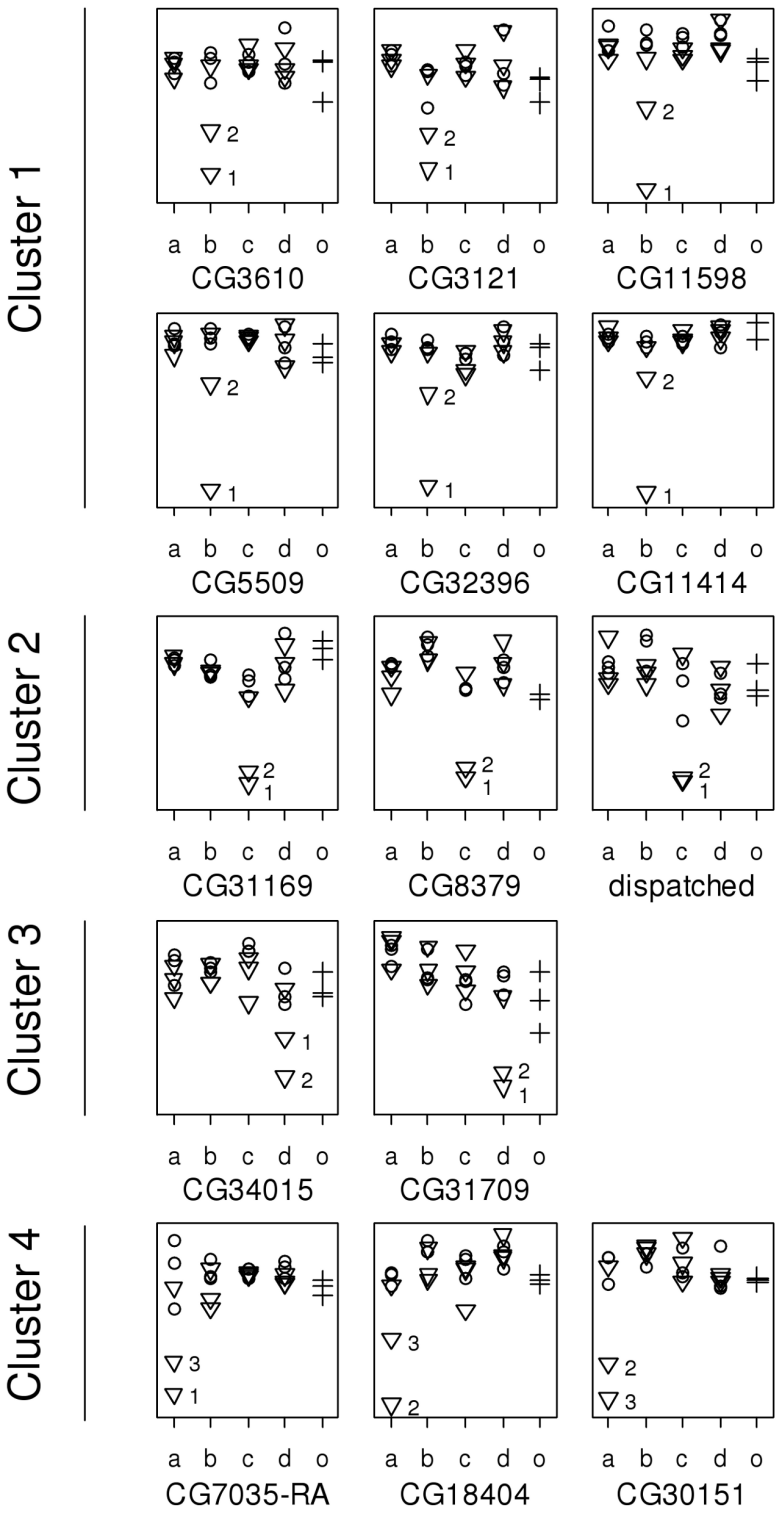
Expression results for the 14 candidate genes in the four inbred lines (*a*, *b*, *c*, and *d*) and outbred controls (*o*). Triangles, circles and plus signs represent the most depressed, least depressed and control samples, respectively. The outlier sublines’ identity is shown besides their symbol.

A cluster dendrogram of the 14 genes showing a signature of depression-causing changes in expression identified four groups (Figure 5), which corresponded to the lines in which the outliers were found (clusters 1–4 with 6, 3, 2 and 3 genes found in lines *b*, *c*, *d* and *a*, respectively). Figure 6 shows the expression results of the 14 genes. For the six genes in cluster 1 the two outlier sublines of line *b* were always the same and appeared in the same order (see sublines numbered 1 and 2 and identified by triangles in Fig. 6). This is consistent with a common regulation for the six genes. Table 1 shows the pairwise linear correlations between the expressions of these 6 genes before (*r*, above diagonal) and after (*r* ´, below diagonal) removing the two outlier sublines with extreme differential expression. The average correlation was *r* = 0.881 considering all sublines and *r* ´ = 0.468 after removal of the two outlier sublines. Thus, the correlation was not generated only by the two outliers, providing additional evidence for common regulation among at least 5 of the 6 genes (note that *r* ´ tended to be low and not significant for CG11414).

**Table 1:** Correlation between the expressions of the 6 genes of cluster 1.

	CG3610	CG3121	CG11598	CG5509	CG32396	CG11414
CG3610	–	**0.921**	**0.872**	**0.901**	**0.886**	**0.842**
CG3121	**0.770**	–	**0.815**	**0.846**	**0.832**	**0.769**
CG11598	**0.445**	0.418	–	**0.906**	**0.935**	**0.891**
CG5509	**0.690**	**0.661**	0.432	–	**0.923**	**0.933**
CG32396	**0.565**	**0.548**	**0.616**	0.409	–	**0.943**
CG11414	0.313	0.297	0.164	0.226	**0.462**	–

Above diagonal (*r*): calculated with all 27 samples; below diagonal (*ŕ*): calculated with 25 samples (two outlier sublines removed from the calculations). In bold, significant values (*t* test, 23 d.f., *P*>0.05) after the Benjamini and Hochberg’s [57] multitesting correction as applied by the p.adjust R function.

Common regulation was less obvious in the remaining clusters. For cluster 2 there was complete coincidence in both subline and order for the outliers (Fig. 6), but the average *r* ´ was only 0.088. For cluster 3, the outlier sublines coincided but their order changed and *r* ´ was 0.061. For cluster 4, two genes shared their outlier sublines, but in reverse order, and the third gene had one of its outliers in a subline which was not outlier for the other two genes. The average value of *r* ´ for this cluster was 0.010 (we removed three sublines instead of two to calculate *r* ´ here, as the outliers were in three sublines for this cluster).

The ontology information for the 14 genes can be seen in Table 2. The DAVID analysis could not find any functionally related genes for any of them (no gene passed the DAVID’s default threshold of 0.25 for the kappa statistic measuring the degree of sharing of annotation terms between genes). No general patterns were found, although microtubule cytoskeleton terms appeared in two genes of cluster 1.

**Table 2:** GO ontology for the 14 genes showing a signature of expression changes generating inbreeding depression.

CLUSTER	AFFYID	GENE ID (CYTOLOGICAL POSITION)	CH		CATEGORY	GO TERM
1	1632745_at	CG3610 (88D5–88D5)	3R	–		
	1633263_at	CG3121(60B3–60B3)	2R	GOTERM_BP GOTERM_CC GOTERM_MF INTERPRO		microtubule-based process, cytoskeleton, microtubule associated complex, microtubule binding, cytoskeletal protein binding, tubulin binding, radial spokehead-like protein
	1627716_at	CG11598 (87C3–87C3)	3R	GOTERM_MF INTERPRO		carboxylesterase activity, triacylglycerol lipase activity, lipase activity, Alpha/beta hydrolase fold-1, AB-hydrolase associated lipase region
	1632716_at	CG5509 (87B11–87B11)	3R	**–**		
	1628979_at	Probable tubulin beta chain CG32396 (65B4–65B4)	3L	GOTERM_BP GOTERM_CC GOTERM_MF INTERPRO		protein complex assembly, microtubule-based process, cytoskeleton, microtubule, microtubule cytoskeleton, nucleotide binding, GTPase activity, structural molecule activity, tubulin, beta tubulin, tubulin/FtsZ, GTPase domain
	1631406_at	CG11414 (60D5–60D5)	2R	GOTERM_MF INTERPRO		zinc ion binding, ion binding, cation binding, zinc finger, RING-type, zinc finger, C2H2-type
**2**	1636918_a_at	CG31169 (94A6–94A11)	3R	GOTERM_BP GOTERM_CC GOTERM_MF INTERPRO		glycerol metabolic process, glycerol–3-phosphate metabolic process, glycerol–3-phosphate dehydrogenase complex, nucleotide binding, DNA binding, BESS motif, NAD-dependent glycerol–3-phosphate dehydrogenase
	1633784_at	CG8379 (85B2–85B2)	3R	INTERPRO		region of unknown function DUF1741
	1635021_at	dispatched CG2019 (83C5–83C5)	3R	GOTERM_BP GOTERM_CC INTERPRO		exocytosis, cell motion, cell surface receptor linked signal transduction, integral to membrane, intrinsic to membrane, sterol-sensing 5TM box
**3**	1632373_s_at	CG34015 (14C6–14C6)	X	INTERPRO		histidine triad (HIT) protein, histidine triad motif
	1640759_at	CG31709 (30B12–30B12)	2L	**–**		
**4**	1634789_at	CG30151 (57A4–57A4)	2R	**–**		
	1636071_a_at	cap binding protein 80 CG7035-RA (4C11–4C11)	X	GOTERM_BP GOTERM_CC GOTERM_MF		RNA splicing, via transesterification reactions, nuclear mRNA splicing, via spliceosome, nuclear cap binding complex, RNA cap binding complex, RNA cap binding, RNA binding
	1627895_at	CG18404 (99E3–99E3)	3R	**–**		

They are shown in the same order as in the dendrogram of Fig 5. No ontological information was found for five of the genes.

In order to evaluate the impact of SNPs on probe hybridization, we looked at match probes within the Affymetrix Drosophila Genome 2.0 array and compared them against the cDNA sequences from transcripts to see which samples contained SNPs within them. For this analysis, two outbred controls and a subset of inbred sublines were sequenced for seven candidate genes. These included the six genes of cluster 1 and one gene of cluster 3 (Table 3). For the former, the most depressed sublines analysed were those showing a significant change in expression (sublines 1 and 2 of line *b* in cluster 1; see Fig. 6). For gene CG34015 of cluster 3, the most depressed subline was subline 2 of line *d*, that showing the largest change in expression for this gene (see Fig. 6). For all genes, two of the least depressed sublines analysed corresponded to line *b* and one to line *d*.

**Table 3:** Distribution of sequence mismatches to array probe sets.

Gene/Sample	No. samples	Polym.*	Polym. **	Fixed differences
**CG3610**				
+D	2	0	0	0
−D	2	0	0	0
Controls	2	0	0	0
All	6	0	0	0
**CG3121**				
+D	2	4(1)	0	0
−D	2	0	0	0
Controls	2	4(1)	0	0
All	6	4(1)	0	0
**CG11598**				
+D	1	0	0	1
−D	2	3(2)	1	0
Controls	2	0	0	0
All	5	3(2)	1	0
**CG5509**				
+D	0	–	–	–
−D	2	0	0	0
Controls	2	0	0	0
All	4	0	0	0
**CG32396**				
+D	2	1	1	0
−D	2	0	0	0
Controls	2	0	0	0
All	6	1	1	0
**CG11414**				
+D	1	0	0	1
−D	2	0	0	1
Controls	2	0	0	2(1)
All	5	1(1)	1(1)	1
**CG34015**				
+D	1	0	0	0
−D	1	0	0	0
Controls	2	0	0	0
All	4	0	0	0

The first column shows the gene and sample analyzed (+D: most depressed; −D: least depressed, All: +D, −D and controls). The next four columns indicate the number of samples, sites that are polymorphic using a relaxed criterion* (SNPs supported by one sequenced strand), sites that are polymorphic using a strict criterion** (SNPs supported by the two sequenced strands), and the number of fixed nucleotide differences to each microarray probe set. The number of non-synonymous changes out of the total is indicated in parenthesis.

Table 3 shows the number of polymorphic sites per gene and sample, and the number of fixed differences to each Affymetrix oligonucleotide probe set. Sequence variation in the cDNA fragment encompassing the array oligonucleotide probes was almost negligible. Only 3 SNPs resulting in mismatches with array probes were detected for the total of seven candidate genes. Therefore, mismatches occurred in only one of the 14 probes representing a gene transcript in the array. This pattern did not change substantially even when including in the analysis doubtful SNPs supported by just one (but not two) of the sequenced strands (Table 3). The results above allow us to reject the hypothesis that sequence mismatches could produce misleading gene expression measurements in our data, a result predicted *a priori* given the low levels of genome-wide variation expected in highly inbred lines as those used here. This is further supported by several lines of evidence. First, Affymetrix expression values are calculated as the difference of hybridization signals between a “match” probe and a “mismatch” probe differing by one single nucleotide. If the sample contains one SNP leading to a single mismatch with the match probe, then two mismatches should occur with the corresponding mismatch probe. Thus, the match-mismatch difference should still reveal an expression signal higher than background noise. Further, the expression level of a gene is calculated by taking a robust average (Tukey biweight) of the signals from all probes included in a probe set, thus minimizing the effect of sequence divergence at a single probe.

The newly generated sequence data were also used to evaluate the occurrence of line-specific DNA differences that could be putatively related to the original causes of depression. However, DNA sequence data indicated a lack of differences between the most and least depressed samples, and that inbred samples were very similar to outbred controls (Table S3). Indeed, only three of the seven surveyed genes displayed nucleotide variation in our samples for the assayed DNA fragments. No more than one or two SNPs were detected per gene and, although they were mostly strain-exclusive, no fixed nucleotide differences were observed between the most and least depressed sublines. The comparison between inbred and control samples revealed one and two fixed differences for genes CG11414 and CG34015 respectively (Appendix Table A3). Interestingly, the fixed difference at CG11414 corresponds to a nonsynonymous change of the amino acid alanine by threonine at position 2763 of the GenBank reference sequence NM138088, perhaps as a functional response to overall inbreeding depression. In summary, no evidence of line-specific DNA differences was found, likely as a result of DNA changes responsible for inbreeding depression being located on regulatory sequences rather than on coding regions.

## Discussion

We have identified fourteen transcripts showing the patterns of variation predicted for the causes of inbreeding depression by population genetics models. These fourteen changes in expression are consistent, first, with genetic differences rare or at least at moderately low frequencies in the base population, because they were detected only in one line of the experiment; and second, with deleterious effects, because the excess of coincidences in line and depression level was generated by the most depressed sublines. To be detected in this experiment, genetic effects had to fulfil two additional requirements. First, they had to be large, so that the sublines carrying them had a high probability of being among the most depressed ones. Second, they had to cause substantial changes in transcription levels, so as to be detected in an experimental setting with limited statistical power.

The presence of large, deleterious genetic effects on the fitness of *Drosophila melanogaster* populations is not unexpected. Mutation-accumulation experiments have shown that deleterious mutations of moderately large effect and partially recessive gene action occur frequently [27]–[29]. In addition, experiments using balancing stocks to assay the viability of individuals homozygous for entire chromosomes sampled from field populations of this species detected many rare, recessive and highly deleterious mutations [45]–[48], and Vermeulen *et al*. [49] found evidence for a major QTL generating inbreeding depression. In fact, it has been estimated that approximately half the inbreeding load in *Drosophila melanogaster* is due to lethal genes [22]; [50]. Individual genes making large contributions to inbreeding depression have also been found in other species [51].

Our objective was to search for an association between genes showing large expression changes under inbreeding and substantial inbreeding depression effects on fitness. The finding of an excess of line-specific, marked changes in expression in the most depressed sublines for at least 14 genes is very suggestive because, as explained in the Introduction, changes in expression level can be important drivers of phenotypic variation and therefore of inbreeding depression. However, it must be stressed that our results are of a correlative nature and do not prove that the observed reductions in expression are directly contributing to the depression. Thus, while we cannot assure that the original genetic alterations resulting in the down regulation of these 14 genes have a direct cause-effect relationship with the observed depression, we show that it is possible to find transcription changes showing the properties predicted by current Population Genetics models of inbreeding depression.

We have not a precise estimate of the number of genetic alterations responsible for the large changes in expression observed in the 14 candidate genes. As seen in the Results section, it is likely that the expression changes in the cluster 4 were due to independent genetic alterations that occurred by chance in the same experimental line, whereas the case for common regulation was stronger for the genes of cluster 1. The considerable correlation between the expression patterns of at least five of the six genes from this cluster (Table 1) would be evidence of *trans* regulation, because the expression of these genes was clearly coordinated despite their disperse chromosome locations (2R, 3R, 3L; Table 2). Alternatively, if the six genes from cluster 1 were under the control of six independent regulatory sequences, then it would be necessary to assume 1) that these regulatory sequences were randomly fixed in the same two most depressed sublines of line *a,* and 2) that they also became fixed in many of the remaining 25 sublines, since these genes tended to be correlated even after removing the outlier sublines (Table 1). Thus, the possibility of a random fixation of six independent regulatory sequences with a similar effect on gene expression in each one of the 27 sublines of the experiment represents a rather unlikely scenario.

Finding evidence for trans-regulation in this species is not unexpected. Ayroles *et al*. [18] also found such evidence when they observed that variation in the third chromosome affected the expression of many genes in other chromosomes. A *trans* regulation mechanism is also supported by the lack of DNA sequence differences between cluster 1′s transcripts, and between transcripts from inbred lines and controls (Table S3). While their relative importance is still unclear, the existence of both *cis* and *trans* mechanisms for the regulation of transcription is well established in *Drosophila melanogaster* [52].

None of the 14 transcription outliers were found significantly related with inbreeding in the analyses of Kristensen *et al.* [17]; [53] or were among the three quoted as such by Ayroles *et al.* [18]. Neither were they in the Sørensen’s *et al.* [54] list of genes differentially expressed in *Drosophila melanogaster* selected to withstand different sources of stress. This is consistent with the view that transcription changes common to all inbred individuals are likely responses to the depression, instead of its potential genetic causes. The amount of information provided by this observation is limited, however, given the modest number of genes in these lists relative to the number of genes included in *Drosophila melanogaster*’s microarrays.

Despite their common regulation in our experiment, there was no obvious relationship between the functions of the five transcripts of cluster 1, with the exception of CG32396 and CG3121, both related with microtubules and cytoskeleton (Table 2). No relationships were apparent either for the remaining seven annotated candidate transcripts. Therefore, our experiment did not find any metabolic pathway specifically involved in inbreeding depression. The heterogeneity in the functions of the identified transcript candidates contributing to the depression was consistent with theoretical expectations: the deleterious load in populations would be due to random, unrelated mutations segregating at low frequency and affecting diverse gene functions. A more complete functional annotation of the *Drosophila* genome and more reliable models of gene network regulation will make it possible a more phenotypically relevant functional interpretation of correlational results such as those obtained in the present experiment.

## Supporting Information

Table S1 **Primers and annealing temperature.** PCR cycling conditions were as follows: an initial denaturation step at 94°C for 2 min, then 35 cycles of 96°C for 10 sec, annealing temperature of the corresponding primer set (55–61°C) for 10 sec, and 70°C for 1 min. The PCR was ended with a 10-min incubation step at 70°C.
(DOC)

Table S2 **Analysis of the number of probe sets having two SL-LD outliers.** It is shown, for each direction of change in expression (up or down regulation), the number of these probe sets observed (Obs); the average number after randomizing the inbred subline codes in the whole series of probe sets (10000 randomizations; Exp); the proportion of these randomizations (P rand) reaching numbers as extreme as the observed, and the percentage of the most depressed cases among the observed SL-LD outliers. All: total number of probe sets with two outliers, SL-DL or not.
(DOC)

Table S3 **Distribution of variable sites in pairwise comparisons.** The first column gives the items compared (+D: most depressed sublines;–D: least depressed sublines; D: all depressed sublines). The next six columns indicate the number of samples compared of item 1 and 2 (each item appearing in the same order as referred in the first column), sites that are polymorphic in only one of the items compared (exclusive polymorphism), sites that are polymorphic in both items (shared polymorphisms), and number of fixed nucleotide differences. The number of non-synonymous changes out of the total is indicated in parenthesis.
(DOC)
