# Supplementary material for: Candidate Transcriptomic Sources of Inbreeding Depression in Drosophila melanogaster
Source: PLoS One. 2013 Jul 29;8(7):e70067. doi: 10.1371/journal.pone.0070067 (PMC3726430; doi:10.1371/journal.pone.0070067)
Supplement: Table S1 — Primers and annealing temperature. PCR cycling conditions were as follows: an initial denaturation step at 94°C for 2 min, then 35 cycles of 96°C for 10 sec, annealing temperature of the corresponding primer set (55–61°C) for 10 sec, and 70°C for 1 min. The PCR was ended with a 10-min incubation step at 70°C. (DOC) [file pone.0070067.s001.doc]

Table S1. Primers and annealing temperature.

| **Locus** | **Primer name** | **Sequence** | **T_a_(°C)** |
| --- | --- | --- | --- |
| ***CG3610*** | CG3610-D | 5´- ACGGCGGCCTATGTGTCAAAG | 58 |
|  | CG3610-R | 5´- CCTTAATACGGGCATCTAGCG |  |
| ***CG3121*** | CG3121-D | 5´- AAGGCCGCGTTGTCTGGTAC | 61 |
|  | CG3121-R | 5´- GATCGGCGAACTCGTCATCA |  |
| ***CG11598*** | CG11598-D | 5´- CGGAGTACAACAAGTTGGTCAAG | 55 |
|  | CG11598-R | 5´- ATTCGTTTATGAAGTGAAAGGTTAT |  |
| ***CG5509*** | CG5509-D | 5´- GATGCTCGCACCTACGGATT | 55 |
|  | CG5509-R | 5´- TCCAGTCCATTTTTGCCTTTG |  |
| ***CG32396*** | CG32396-D | 5´- CGGCTGCACTTCCTCATACC | 55 |
|  | CG32396-R | 5´- TTCCAAGACGCGTAATTACA |  |
| ***CG11414*** | CG11414-D | 5´- GCTGGGCGAGTCGTATGACATT | 58 |
|  | CG11414-R | 5´- TGCGGGCATTTGCTAGACACA |  |
| ***CG34015*** | CG34015-D | 5´- GCGGACGACAACTGCATT | 55 |
|  | CG34015-R | 5´- GGCCTCTGACAGGGGGTAAAC |  |
