# Supplementary material for: Candidate Transcriptomic Sources of Inbreeding Depression in Drosophila melanogaster
Source: PLoS One. 2013 Jul 29;8(7):e70067. doi: 10.1371/journal.pone.0070067 (PMC3726430; doi:10.1371/journal.pone.0070067)
Supplement: Table S2 — Analysis of the number of probe sets having two SL-LD outliers. It is shown, for each direction of change in expression (up or down regulation), the number of these probe sets observed (Obs); the average number after randomizing the inbred subline codes in the whole series of probe sets (10000 randomizations; Exp); the proportion of these randomizations (P rand) reaching numbers as extreme as the observed, and the percentage of the most depressed cases among the observed SL-LD outliers. All: total number of probe sets with two outliers, SL-DL or not. (DOC) [file pone.0070067.s002.doc]

Table S2. Analysis of the number of probe sets having two SL-LD outliers.

| α value | Two outliers up regulated | | | | |  | Two outliers down regulated | | | | |
| --- | --- | --- | --- | --- | --- | --- | --- | --- | --- | --- | --- |
|  | All | SL-DL Obs. | SL-DL Exp. | P rand. | % Most Depr. Obs |  | All | SL-DL Obs | SL-DL Exp | P rand. | % Most Depr. Obs |
| 0.001 | 0 | 0 | - | - | - |  | 6 | 4 | 0.744 | 2 e-4 | 75.0 |
| 0.01 | 9 | 0 | 1.115 | 0.337 | - |  | 21 | 9 | 2.262 | 4 e-4 | 77.8 |
| 0.05 | 28 | 5 | 3.523 | 0.125 | 40.0 |  | 46 | 18 | 5.774 | < 1 e-4 | 77.8 |
| 0.10 | 52 | 9 | 6.401 | 0.126 | 55.6 |  | 71 | 31 | 8.807 | < 1 e-4 | 61.3 |
| 0.15 | 88 | 14 | 10.890 | 0.168 | 64.3 |  | 112 | 36 | 13.992 | < 1 e-4 | 58.3 |
| 0.20 | 112 | 16 | 13.992 | 0.248 | 62.5 |  | 136 | 38 | 16.991 | < 1 e-4 | 55.3 |
| 0.25 | 154 | 25 | 19.677 | 0.276 | 64.0 |  | 168 | 48 | 21.084 | < 1 e-4 | 54.2 |
