# Supplementary material for: Candidate Transcriptomic Sources of Inbreeding Depression in Drosophila melanogaster
Source: PLoS One. 2013 Jul 29;8(7):e70067. doi: 10.1371/journal.pone.0070067 (PMC3726430; doi:10.1371/journal.pone.0070067)
Supplement: Table S3 — Distribution of variable sites in pairwise comparisons. The first column gives the items compared (+D: most depressed sublines;–D: least depressed sublines; D: all depressed sublines). The next six columns indicate the number of samples compared of item 1 and 2 (each item appearing in the same order as referred in the first column), sites that are polymorphic in only one of the items compared (exclusive polymorphism), sites that are polymorphic in both items (shared polymorphisms), and number of fixed nucleotide differences. The number of non-synonymous changes out of the total is indicated in parenthesis. (DOC) [file pone.0070067.s003.doc]

Table S3. Distribution of sequence mismatches to array probe sets.

| Comparison | No. samples | |  | Exclusive polymorphism | | Shared polymorphism | Fixed differences |
| --- | --- | --- | --- | --- | --- | --- | --- |
|  | Item 1 | Item 2 |  | Polym. 1 | Polym. 2 |  |  |
| CG3610 (634 nt) |  |  |  |  |  |  |  |
| +D *vs* –D | 2 | 2 |  | 0 | 0 | 0 | 0 |
| +D *vs* controls | 2 | 2 |  | 0 | 0 | 0 | 0 |
| –D *vs* controls | 2 | 2 |  | 0 | 0 | 0 | 0 |
| D *vs* controls | 4 | 2 |  | 0 | 0 | 0 | 0 |
| CG3121 (511 nt) |  |  |  |  |  |  |  |
| +D *vs* –D | 2 | 2 |  | 1 | 0 | 0 | 0 |
| +D *vs* controls | 2 | 2 |  | 1 | 0 | 0 | 0 |
| –D *vs* controls | 2 | 2 |  | 0 | 0 | 0 | 1 |
| D *vs* controls | 4 | 2 |  | 1 | 0 | 0 | 0 |
| CG11598 (705 nt) |  |  |  |  |  |  |  |
| +D *vs* –D | 1 | 2 |  | 0 | 0 | 0 | 0 |
| +D *vs* controls | 1 | 2 |  | 0 | 0 | 0 | 1 |
| –D *vs* controls | 2 | 2 |  | 0 | 0 | 0 | 0 |
| D *vs* controls | 3 | 2 |  | 0 | 0 | 0 | 0 |
| CG5509 (522 nt) |  |  |  |  |  |  |  |
| +D *vs* –D | 0 | 2 |  | - | - | - | - |
| +D *vs* controls | 0 | 2 |  | - | - | - | - |
| –D *vs* controls | 2 | 2 |  | 0 | 1 | 0 | 0 |
| D *vs* controls | 2 | 2 |  | 0 | 1 | 0 | 0 |
| CG32396 (675 nt) |  |  |  |  |  |  |  |
| +D *vs* –D | 2 | 2 |  | 2 | 0 | 0 | 0 |
| +D *vs* controls | 2 | 2 |  | 1 | 0 | 1 | 0 |
| –D *vs* controls | 2 | 2 |  | 0 | 1 | 0 | 0 |
| D *vs* controls | 4 | 2 |  | 1 | 0 | 1 | 0 |
| CG11414 (686 nt) |  |  |  |  |  |  |  |
| +D *vs* –D | 1 | 2 |  | 0 | 0 | 0 | 0 |
| +D *vs* controls | 1 | 2 |  | 0 | 0 | 0 | 1(1) |
| –D *vs* controls | 2 | 2 |  | 0 | 0 | 0 | 1(1) |
| D *vs* controls | 3 | 2 |  | 0 | 0 | 0 | 1(1) |
| CG34015 (480 nt) |  |  |  |  |  |  |  |
| +D *vs* –D | 1 | 1 |  | 0 | 0 | 0 | 0 |
| +D *vs* controls | 1 | 2 |  | 0 | 0 | 0 | 2 |
| –D *vs* controls | 1 | 2 |  | 0 | 0 | 0 | 2 |
| D *vs* controls | 2 | 2 |  | 0 | 0 | 0 | 2 |
